# A Substitute for Rubber Gloves in Surgery

**Published:** 1905-01

**Authors:** 


### A Substitute for Rubber Gloves in Surgery.

Dr. Frederick Holme Wiggin, of New York, said at the New York State Medical Association, 21st annual meeting, October 17, 1904, (*Medical Record,* October 22, 1904,) there were a great many annoying features connected with the use of rubber gloves in operative work. Attention was called to a substitute which has given him the greatest satisfaction. In the hospital the following was prepared: 49½ ozs. alcohol (96 per cent), 49½ ozs. ether, ½ oz. celloidin and 1 oz. castor oil. The hands of the operator were sterilised and then dipped in this solution. This gave a thick, firm, dry, elastic coating that did not crack and was not soluble in water or ordinary alcohol. It could be removed by washing in equal parts of alcohol and ether.

